# Definitive radiotherapy versus surgery after neoadjuvant immunochemotherapy for locally advanced esophageal squamous cell carcinoma: a propensity score-matched study of efficacy and safety

**DOI:** 10.3389/fonc.2026.1885302

**Published:** 2026-10-12

**Authors:** Qiuying An, Yage Jia, Menglu Liu, Liyuan Fu, Jingxin Bai, Zihan Zhang, Jin Yan, Yajing Wang, Hongshan Ji, Yuhao Su, Ping Zhang, Zhiguo Zhou

**Affiliations:** Department of Radiation Oncology, The Fourth Hospital of Hebei Medical University, Shijiazhuang, Hebei, China

**Keywords:** locally advanced esophageal squamous cell carcinoma, neoadjuvant immunochemotherapy, prognosis, radiotherapy, recurrence, surgery

## Abstract

**Background:**

The optimal local treatment strategy for locally advanced esophageal squamous cell carcinoma (LA-ESCC) following neoadjuvant immunochemotherapy (NICT) remains controversial. This study aimed to compare the survival outcomes and recurrence patterns of radical surgery and definitive radiotherapy (RT) following NICT in patients with LA-ESCC.

**Methods:**

Patients with LA-ESCC who underwent NICT (2019–2023) were enrolled and categorized into surgery or RT groups. Propensity score matching (PSM) and Kaplan-Meier methods were used to estimate progression-free survival (PFS) and overall survival (OS). The differences in recurrence patterns between the two groups were compared.

**Results:**

A total of 278 patients were included, comprising 218 (78.4%) and 60 (21.6%) patients in the surgery and RT groups, respectively. After PSM, the surgery group (n=117) maintained a significant OS benefit over the RT group (n=60) (3-year OS rates: 72.1% vs. 59.5%; *P* = 0.028), whereas the PFS rates were comparable between the two groups (3-year PFS rates: 59.4% vs. 46.9%; *P* = 0.12). For patients with upper esophageal cancer, RT showed a trend toward improved survival (3-year PFS rates: 76.9% vs. 55.5%, *P* = 0.34; 3-year OS rates: 74.0% vs. 61.8%, *P* = 0.50). Among the responders after NICT (near-cCR/cPR), PFS and OS were similar between the surgery and RT groups (3-year PFS rates: 68.9% vs. 66.9%, *P* = 0.64; 3-year OS rates: 80.2% vs. 70.7%, *P* = 0.20). Regional lymph node recurrence predominated in the surgery group (50.0%), whereas locoregional esophageal recurrence was most frequent in the RT group (37.0%). The treatment-related mortality rates were 0.8% (1/117) in the surgery group and 1.7% (1/60) in the RT group, indicating the overall safety of the treatments.

**Conclusions:**

Radical surgery remains the standard treatment for LA-ESCC after NICT. Definitive RT may be an organ-preserving option in selected patients, especially those with upper esophageal cancer or a favorable response to NICT.

## 1 Introduction

Esophageal cancer remains a common malignant tumor of the digestive tract, characterized by a persistently poor 5-year overall survival (OS) rate despite advancements in multimodal therapeutic strategies (1). For patients with locally advanced esophageal squamous cell carcinoma (LA-ESCC), the standard treatment involves neoadjuvant therapy followed by radical esophagectomy (2). However, esophagectomy is a highly invasive procedure associated with significant perioperative morbidity and long-term functional impairments, which profoundly diminish patients’ health-related quality of life (3). In recent years, the application of neoadjuvant immunochemotherapy (NICT) has significantly improved the rates of pathological complete response (pCR) and major pathological remission (MPR) after surgery in patients with LA-ESCC (4). Consequently, these impressive response rates have reignited the debate regarding “organ preservation” strategies in these patients. While the SANO and preSINO trials established the feasibility of “active surveillance” following neoadjuvant chemoradiotherapy (NCRT) (3, 5), the optimal management of patients achieving a clinical complete response (cCR) after NICT remains an unresolved dilemma. Simple surveillance may expose patients who are ineligible for or refuse surgery to a high risk of locoregional recurrence.

Definitive radiotherapy (RT), an important organ-preserving treatment strategy (6, 7), has been found in some retrospective studies to achieve survival rates similar to those of surgery in patients who achieve MPR after neoadjuvant therapy (6). Nevertheless, it remains unclear which patient subgroups benefit from RT, and the specific patterns of recurrence associated with this approach are not well understood (8). Therefore, the present study retrospectively analyzed the survival outcomes and recurrence patterns of patients with LA-ESCC who underwent radical surgery or RT following NICT. This study aimed to optimize organ-preservation strategies in the era of immunotherapy.

## 2 Materials and methods

### 2.1 Patients

This retrospective cohort study collected patients with thoracic esophageal squamous cell carcinoma (ESCC) who underwent NICT at the Fourth Hospital of Hebei Medical University between January 2019 and June 2023. The inclusion criteria were as follows: 1) age 18–75 years; 2) pathological confirmation of thoracic ESCC; 3) clinical stage cT_1-2_N_+_M_0_ or cT_3-4a_N_any_M_0_ according to the 8th edition of the American Joint Committee on Cancer (AJCC) staging system; 4) completion of 2–3 cycles of NICT; and 5) radical surgery or definitive radiotherapy after NICT. The exclusion criteria were as follows: 1) history of other malignancies or multiple primary cancers; 2) diagnosis of unresectable tumors; 3) disease progression during NICT (Response Evaluation Criteria in Response Evaluation Criteria in Solid Tumors1.1); and 4) insufficient clinical or follow-up data. Based on the treatment modality following NICT, patients were categorized into the surgery or RT groups.

### 2.2 Neoadjuvant therapy and efficacy evaluation

Neoadjuvant chemotherapy regimens included taxanes plus platinum administered every 3 weeks in combination with immunotherapy. Immunotherapy uses programed cell death protein 1 (PD-1) inhibitors, including tislelizumab, camrelizumab, sintilimab, toripalimab, and pembrolizumab.

Treatment response was evaluated using computed tomography (CT) scans by measuring the longest axial diameter of the primary esophageal lesion and the shortest diameter of the metastatic lymph nodes. According to the response evaluation criteria for target lesions (9), treatment response was classified as clinical partial response (cPR), clinical stable disease (cSD), or clinical progressive disease (cPD). We further defined clinical near-complete response (near-cCR) among patients who achieved cPR. Near-cCR was assessed based on CT findings and defined as an axial long axis of the primary esophageal lesion of less than 5 mm on CT and a short-axis diameter of metastatic lymph nodes of less than 5 mm.

### 2.3 Surgery and radiotherapy after NICT

Treatment options for the patient after NICT are determined through multidisciplinary evaluation and collaborative decision-making. Surgery is recommended for patients with acceptable surgical risk who are willing to undergo esophagectomy, whereas definitive RT is considered for patients with medically high surgical risk, or those who refuse surgery and wish to preserve their organs.

For patients who underwent surgery after NICT, radical esophagectomy was performed with two- or three-field lymph node dissections. Postoperative adjuvant therapy was not mandatory but was recommended for patients who did not achieve pCR or whose preoperative staging was cT_3-4a_/cN_+_.

Definitive RT was delivered with intensity-modulated radiotherapy or helical tomotherapy using 6-MV X-rays, based on involved-field irradiation. The gross tumor volume (GTV) comprised the primary tumor (GTVp) and clinically involved regional lymph nodes (GTVn). The clinical target volume (CTV) for GTVp extended 2–3 cm longitudinally and 0.5–1.0 cm radially, whereas GTVn was expanded uniformly by 0.5–1.0 cm. The planning target volume (PTV) was defined as the CTV plus a uniform 0.5–1.0 cm margin. The prescribed dose ranged from 50.4 to 64 Gy, with a fraction dose of 1.8–2.1 Gy. Dose constraints for organs at risk included a mean lung dose <15 Gy and V20 <25% for the lung, V30 <40% and V40 <30% for the heart, and a maximum spinal cord dose <45 Gy.

### 2.4 Follow-up and outcomes

Follow-up was conducted via outpatient visits, hospitalization records, and telephone interviews, with the final follow-up being censored on June 30, 2025. OS was defined as the interval from the initiation of treatment to death or the last follow-up. Progression-free survival (PFS) was defined as the time from treatment initiation to disease progression, recurrence, metastasis, or last follow-up.

Recurrence patterns included local recurrence (LR), distant recurrence (DR), and local recurrence with distant metastasis (LR+DR). LR was defined as recurrence in the lymph nodes of the esophageal stations, recurrence in the esophageal tumor bed and anastomosis site after surgery, or primary recurrence in the esophageal region after RT. DR was defined as extra-regional lymph nodes or hematogenous metastases. Surgical complications were graded according to the Clavien-Dindo classification, and radiation-related toxicities were assessed using the Common Terminology Criteria for Adverse Events (CTCAE) version 5.0.

### 2.5 Statistical analysis

Statistical analysis were performed using SPSS version 26.0 and R version 4.1. Continuous variables are reported as medians with interquartile ranges (Interquartile Range: IQR). Categorical variables were compared using the chi-square test or Fisher’s exact test. Propensity score matching (PSM) was performed at a 2:1ratio between the surgery and RT groups using the nearest-neighbor algorithm without replacement, with a caliper width of 0.2.Matching variables included age, tumor location, tumor length, cT stage, cN stage, and response to NICT. The standardized mean differences before and after matching are shown in **Supplementary Table S1**. Survival curves for OS and PFS were estimated using the Kaplan-Meier method and compared using the log-rank test. Subgroup analysis were conducted using an unstratified Cox proportional hazards model, including treatment as the primary covariate. A two-sided *P-*value < 0.05 was considered statistically significant.

## 3 Results

### 3.1 Clinical characteristics of patients

The entire cohort comprised 278 patients, including 191 men and 87 women. The median age was 65 years (IQR: 59–69 years), the median tumor length was 5 cm (IQR: 4–7 cm), and the median body mass index (BMI) was 23.15 (IQR: 21.00–25.29). After NICT, 74 patients (26.6%) achieved near-cCR, and 97 patients (34.9%) achieved cPR.

### 3.2 The treatments for the patients after NICT

After NICT, 218 patients (78.4%) underwent radical surgery (surgery group), and 60 patients (27.5%) received definitive radiotherapy (RT group). As shown in **Table 1**, patients who achieved near-cCR or cPR were more likely to undergo surgery than radiotherapy (65.6% vs. 46.7%, *P* = 0.008).

**Table 1:** Comparison of clinical characteristics between the surgery and RT groups before and after PSM.

Characteristics	Before PSM		*P*-Value	After PSM		*P*-Value
	Surgery (n=218, %)	RT (n=60, %)		Surgery (n=117, %)	RT (n=60, %)	
Age			0.108			0.974
≤65	120 (55)	26 (43.3)		51 (43.6)	26 (43.3)	
>65	98 (45)	34 (56.7)		66 (56.4)	34 (56.7)	
Sex			0.311			0.162
Male	153 (70.2)	38 (63.3)		86 (73.5)	38 (63.3)	
Female	65 (29.8)	22 (36.7)		31 (26.5)	22 (36.7)	
BMI			1.000			0.957
≤23.15	109 (50.0)	30 (50.0)		59 (50.4)	30 (50.0)	
>23.15	109 (50.0)	30 (50.0)		58 (49.6)	30 (50.0)	
Diabetes			0.759			0.814
No	197 (90.4)	55 (91.7)		106 (90.6)	55 (91.7)	
Yes	21 (9.6)	5 (8.3)		11 (9.4)	5 (8.3)	
Family history			0.364			0.554
No	162 (74.3)	48 (80.0)		89 (76.1)	48 (80.0)	
Yes	56 (25.7)	12 (20.0)		28 (23.9)	12 (20.0)	
Tumor location			0.168			0.113
Ut	30 (13.8)	13 (21.7)		17 (14.5)	13 (21.7)	
Mt	82 (37.6)	25 (41.7)		38 (32.5)	25 (41.7)	
Lt	106 (48.6)	22 (36.7)		62 (53.0)	22 (36.7)	
Tumor length			0.676			0.953
≤5cm	112 (51.4)	29 (48.3)		56 (47.9)	29 (48.3)	
>5cm	106 (48.6)	31 (51.7)		61 (52.1)	31 (51.7)	
cT stage			0.767			0.428
T2	32 (14.7)	7 (11.7)		12 (10.3)	7 (11.7)	
T3	178 (81.6)	50 (83.3)		103 (88.0)	50 (83.3)	
T4a	8 (3.7)	3 (5.0)		2 (1.7)	3 (5.0)	
cN stage			0.516			0.691
N0	101 (46.3)	23 (38.3)		51 (43.6)	23 (38.3)	
N1	79 (36.2)	26 (43.3)		43 (36.8)	26 (43.3)	
N2-3	38 (17.5)	11 (18.4)		23 (19.6)	11 (18.4)	
cTNM stage			0.249			0.789
II	112 (56.0)	27 (45.0)		59 (50.4)	27 (45.0)	
III	83 (38.0)	30 (50.0)		53 (45.3)	30 (50.0)	
IVa	13 (6.0)	3 (5.0)		5 (4.3)	3 (5.0)	
Cycles of NICT			0.959			0.895
2	130 (59.6)	36 (60.0)		69 (59.0)	36 (60.0)	
3	88 (40.4)	24 (40.0)		48 (41.0)	24 (40.0)	
Response to NICT			0.014			0.945
near-cCR	65 (29.8)	9 (15.0)		17 (14.5)	9 (15.0)	
cPR	78 (35.8)	19 (31.7)		40 (34.2)	19 (31.7)	
cSD	75 (34.4)	32 (53.3)		60 (51.3)	32 (53.3)	

BMI, Body mass index; Ut, upper esophageal cancer; Mt, Middle esophageal cancer; Lt, Lower esophageal cancer; near-cCR; Clinical near-complete response; cPR, Clinical partial response; cSD, Clinical stable disease.

In the surgery group, the R0 resection rate was 94.0% (205/218) and the pCR rate was 29.4% (64/218). Among patients in the RT group, a total of three patients (5.0%) interrupted treatment, including one patient who died from an esophageal-tracheal fistula after receiving 32 Gy of radiotherapy and two patients who experienced grade III radiation esophagitis during treatment and refused to continue radiotherapy, with doses of 43.4 Gy and 48 Gy, respectively.

### 3.3 Survival analysis

#### 3.3.1 Survival outcomes before PSM

The median follow-up period for the entire cohort was 39.5 months. Before PSM, the median PFS and OS were not reached. The 3-year PFS and OS rates for the entire cohort were 60.6% and 73.5%, respectively. Patients in the surgery group demonstrated superior survival outcomes compared to those in the RT group, exhibiting significantly higher 3-year PFS (64.2% vs. 46.9%, *P* = 0.02) and OS rates (77.3% vs. 59.5%, *P* = 0.001).

#### 3.3.2 Survival outcomes after PSM

After PSM, the baseline characteristics were balanced between the surgery (n=117) and RT (n=60) groups (**Table 1**). Although the 3-year PFS rate remained numerically higher in the surgery group (59.4% vs. 46.9%, *P* = 0.12, **Figure 1A**). However, the surgery group maintained a significant advantage in 3-year OS compared with the RT group (72.1% vs. 59.5%, *P* = 0.028, **Figure 1B**). Subgroup analysis revealed no significant differences in PFS between the two treatment modalities across any of the evaluated subgroups (*P* > 0.05, **Figure 2A**). Regarding OS, surgery conferred a significant benefit in patients aged > 65 years with lower third esophageal cancer at cT3 stage (*P* < 0.05, **Figure 2B**).

**Figure 1:**
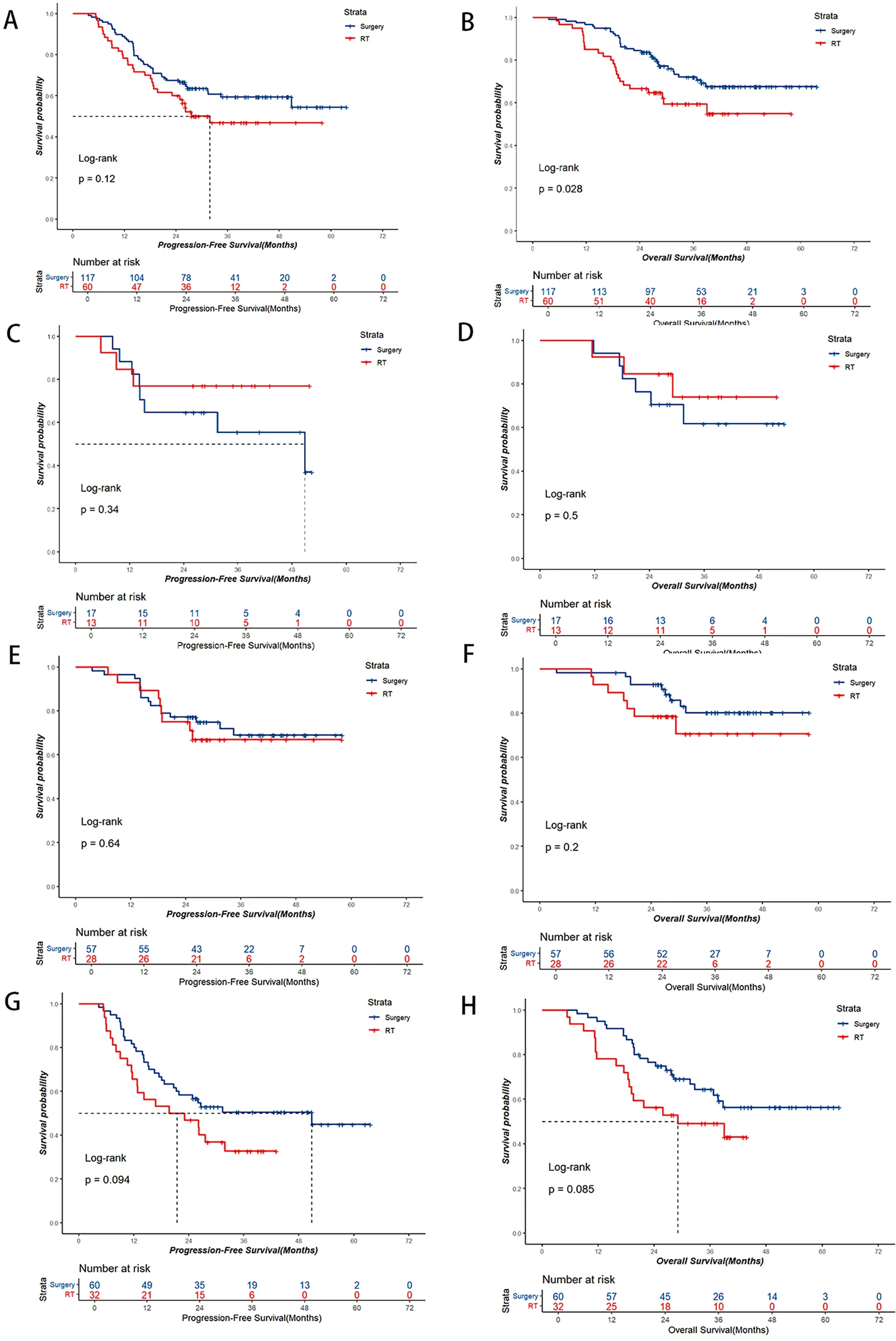
Kaplan-Meier curves of PFS and OS between the surgery and RT groups after PSM. **(A, B)** PFS and OS in all patients.; **(C, D)** PFS and OS in patients with upper esophageal cancer; **(E, F)** PFS and OS in responders; **(G, H)** PFS and OS in non-responders.

**Figure 2:**
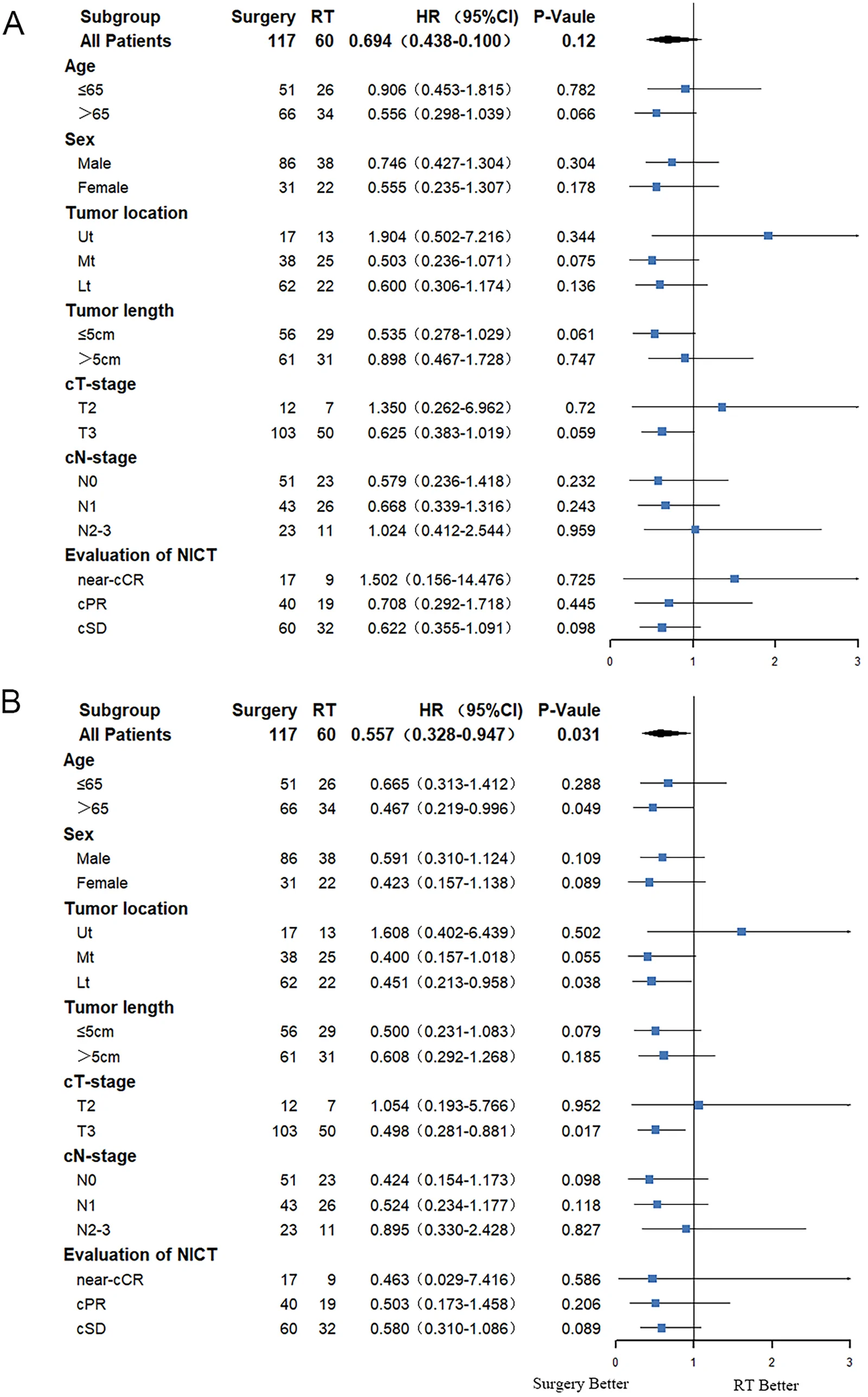
Forest plot of subgroup analysis for PFS and OS. Hazard ratios **(HRs)** and 95% confidence intervals **(CIs)** comparing surgery versus RT across prespecified subgroups for **(A)** PFS and **(B)** OS.

Univariate and multivariate Cox regression analysis were used to identify the factors influencing PFS and OS (**Supplementary Tables S2**, **S3**, respectively). In the multivariate analysis, surgery after NICT was identified as an independent prognostic factor for better OS (HR = 0.514, *P* = 0.019). Furthermore, no response to NICT (cSD) was significantly associated with worse OS and PFS (*P* < 0.05).

### 3.4 Stratified analysis

#### 3.4.1 Tumor location

For patients with upper esophageal cancer, RT showed a trend toward improved survival (3-year PFS rates: 76.9% vs. 55.5%, *P* = 0.34; 3-year OS rates: 74.0% vs. 61.8%, *P* = 0.50; **Figures 1C, D**). Conversely, for tumors located in the middle or lower esophagus, the surgery group demonstrated significantly superior survival outcomes compared to the RT group (3-year PFS rates: 60.1% vs. 37.7%, *P* = 0.02; 3-year OS rates: 73.9% vs. 55.4%, *P* = 0.004).

#### 3.4.2 Clinical response to NICT

Patients were stratified based on their clinical response to NICT into responders (near-cCR/cPR, n=85) and non-responders (cSD, n=92). Among the responders, the 3-year PFS rates were comparable between the surgery and RT groups (68.9% vs. 66.9%, *P* = 0.64, **Figure 1E**). The surgery group exhibited a nearly 10% absolute increase in the 3-year OS rate compared with the RT group (80.2% vs. 70.7%, *P* = 0.20, **Figure 1F**). Among the non-responders, the survival rates were higher in the surgery group than in the RT group (3-year PFS rates: 50.5% vs. 32.7%, *P* = 0.094, **Figure 1G**; 3-year OS rates: 64.4% vs. 49.2%, *P* = 0.085, **Figure 1H**).

### 3.5 Recurrence patterns

There was no significant difference in the overall recurrence pattern between the surgery and RT groups, with LR being the most common (**Table 2**). When stratified by clinical response to NICT, non-responders accounted for the vast majority of recurrences in both the surgery (68.2%, 30/44) and RT (66.7%, 18/27) groups (**Figures 3A–C**). Among the non-responders, LR was the predominant pattern in the two groups (53.3% vs. 77.8%, *P* = 0.090, **Figure 3D**). Conversely, in the responders, the local recurrence rate in the surgery group was slightly higher than that in the RT group (64.3% vs. 22.2%, *P* = 0.089, **Figure 3E**), and the RT group had the highest proportion of DR (44.5%).

**Table 2:** Analysis of recurrence patterns in surgery and RT groups.

Recurrence patterns	Surgery group (n=44,%)	RT group (n=27, %)	*P*-Value
**Local recurrence (LR)**	**25 (56.8)**	**16 (59.2)**	**0.840**
Anastomotic stoma	3 (6.8)	--	
Locoregional esophageal	--	10 (37.0)	
Regional LN	22 (50.0)	5 (18.5)	
Locoregional esophageal + Regional LN	--	1 (3.7)	
**Distant metastasis (DR)**	**15 (34.1)**	**8 (29.6)**	**0.697**
Lung	6 (13.7)	1 (3.7)	
Liver	4 (9.1)	2 (7.4)	
Bone	2 (4.5)	4 (14.8)	
Brain	1 (2.3)	--	
Extra-regional LN	2 (4.5)	1 (3.7)	
**LR+DR**	**4 (9.1)**	**3 (11.1)**	**1.000**
Regional LN + Liver	1 (2.3)	--	
Regional LN +Extra-regional LN	3 (6.8)	2 (7.4)	
Locoregional esophageal + Extra-regional LN	--	1 (3.7)	

LN, Lymph node.

Bold values indicate summary categories for the total number of severe treatment-related adverse events, including surgical complications graded according to the Clavien–Dindo classification (Grade III/IV and V) and radiotherapy-related adverse events graded according to CTCAE version 5.0 (Grade 3–4 and 5).

**Figure 3:**
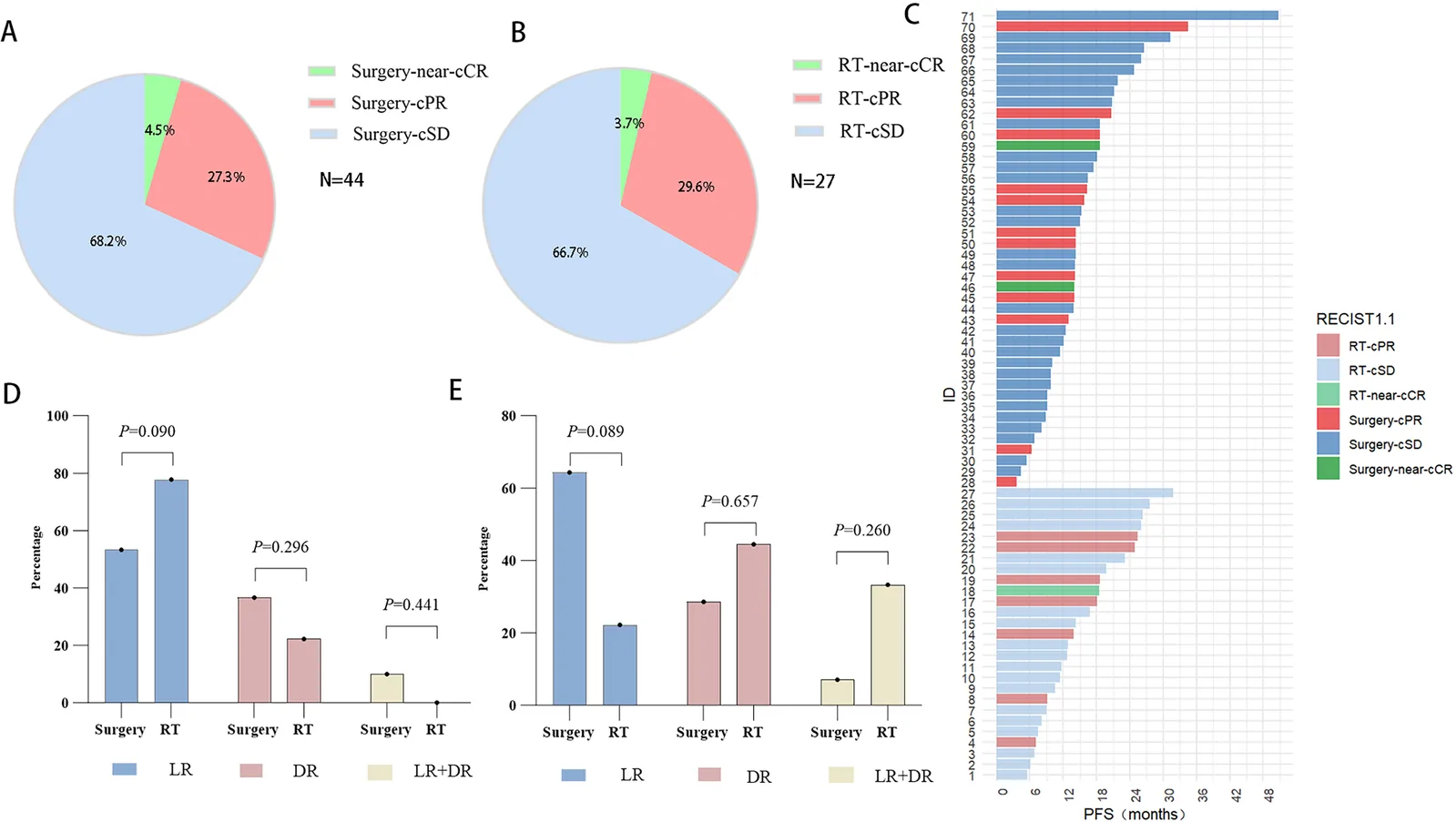
**(A, B)** Distribution of NICT clinical response categories among patients with confirmed recurrence in the surgery group **(A)** and RT group **(B)**. **(C)** PFS analysis specifically for the 71 patients who developed recurrence, stratified by NICT response and treatment modalities. **(D, E)** Comparison of patterns of recurrence between the surgery and RT groups, stratified by NICT response: **(D)** non-responders; **(E)** responders.

### 3.6 Safety of surgery or RT after NICT

In the surgery group, 35.9% (42/117) of patients experienced grade III or higher complications (Clavien-Dindo classification), of which one patient (0.9%) died due to postoperative anastomotic leakage. In the RT group, grade ≥3 adverse events were reported in 46 of 60 patients (76.7%), with one fatality (1.7%) attributed to an esophageal fistula. A detailed summary of all complications and adverse events is presented in **Table 3**.

**Table 3:** Treatment-related adverse events during surgery or RT phase.

TRAEs of surgery (Clavien-Dindo classification)	N=117(%)	TRAEs of radiotherapy (CTCAE 5.0)	N=60(%)
**Total complications (Grade III/IV)**	**41 (35.0)**	**Total Adverse events (Grade 3-4)**	**45 (75.0)**
Pneumonitis	21 (17.9)	Lymphopenia	35 (58.3)
Pneumothorax	4 (3.4)	Myelosuppression	11 (18.3)
Respiratory failure	4 (3.4)	Esophagitis	5 (8.3)
Atelectasis	1 (0.9)	Pneumonitis	2 (3.3)
Pleural effusion	13 (11.1)	**Total Adverse events (Grade 5)**	**1 (1.7)**
Anastomotic leakage	2 (1.7)	Esophageal fistula	1 (1.7)
Wound infections	2 (1.7)		
**Total complications (Grade V)**	**1 (0.9)**		
Anastomotic leakage	1 (0.9)		

Bold values indicate summary categories for the total number of severe treatment-related adverse events, including surgical complications graded according to the Clavien–Dindo classification (Grade III/IV and V) and radiotherapy-related adverse events graded according to CTCAE version 5.0 (Grade 3–4 and 5).

## 4 Discussion

Esophageal cancer remains a major health burden and ranks as the seventh leading cause of cancer-related mortality worldwide (10). In China, ESCC is the predominant histological subtype, with most patients diagnosed at a locally advanced stage. Neoadjuvant combination therapies have demonstrated impressive rates of pCR and MPR in locally advanced disease (4), with pCR serving as an important indicator of favorable long-term prognosis (11). Consequently, trials such as preSINO and SANO have investigated the feasibility of “active surveillance” strategies within the context of neoadjuvant chemoradiotherapy^[3.5]^. However, in the era of immunotherapy, for patients who achieved clinical remission or were medically unfit for surgery following NICT, there is currently a lack of randomized controlled evidence on whether to use esophagectomy or try RT as an organ-preserving alternative. Therefore, this study aimed to evaluate the feasibility of definitive RT as an organ-preserving alternative and identify potential beneficiary subgroups, thereby providing real-world evidence to optimize treatment strategies for NICT.

The results of this study showed that patients who underwent surgery demonstrated superior survival outcomes compared to those treated with definitive RT after NICT. After PSM, the 3-year OS rates of the surgery group were still significantly higher than those of the RT group (72.1% vs. 59.5%, *P* = 0.028), whereas the difference in 3-year PFS was not significant (59.4% vs. 46.9%, *P* = 0.12). The observation of “comparable PFS but divergent OS” likely stems from distinct patterns of failure and the feasibility of subsequent salvage interventions. The recurrence mode of patients in the surgery group predominantly involved regional lymph nodes (50.0%), which could remain candidates for effective salvage strategies, such as radiotherapy or chemoradiotherapy, which can significantly extend post-recurrence survival (12). Conversely, the RT group exhibited a high rate of locoregional esophageal recurrence (37.0%). The treatment modalities for such failures are limited, as the efficacy and safety profiles of re-irradiation or salvage esophagectomy remain ill-defined (13), potentially compromising the overall survival outcomes. Previously, Song et al. (6) conducted a retrospective analysis of 388 LA-ESCC patients who received NICT, of whom 89 received RT and 299 underwent surgery. In their cohort, although the 2-year PFS was significantly better in the surgery group than in the RT group (66.5% vs. 49.6%, *P* = 0.017), no significant difference was observed in the 2-year OS (75.6% vs. 69.1%, *P* = 0.241). Notably, the 2-year OS rate in our surgical cohort (84.5%) was higher than that reported by Song et al. We postulated that this discrepancy was attributable to variations in the inclusion criteria of the studies. Song et al. (6) included patients with supraclavicular lymph node metastases and cervical esophageal cancer. Surgical management of these cases is technically more demanding and associated with poorer prognoses, which may have diluted the survival benefit of surgery in their analysis.

Response-guided strategies are important stratification factors in the individualized treatment of esophageal cancer (6, 7). Our stratified analysis revealed that for responders, the 3-year PFS and OS rates were similar between the surgery and RT groups. This aligns with the recent findings of Song et al. (6). Similarly, in the era of neoadjuvant chemotherapy (NCT), Kiyoshi et al. reported a 5-year survival rate of 77.5% for responders treated with definitive chemoradiotherapy, which was non-inferior to surgical outcomes (7). Conversely, for non-responders (cSD), surgery was associated with better survival outcomes, with both 3-year PFS and OS rates exceeding those of the RT group by more than 12%. Consistent with this finding, Kiyoshi et al. (7) observed that non-responders after NCT had poor outcomes with definitive chemoradiotherapy, with a 5-year OS rate of only 22.3%. This may partly reflect underlying tumor heterogeneity and potential immune evasion, as hypoxic regions, dense stromal barriers, and poorly T-cell-infiltrated tumor microenvironments have been reported to limit drug delivery and T-cell infiltration and to be associated with radioresistance (14–16). Thus, non-responders may be less sensitive to chemotherapy, immunotherapy, and radiotherapy. However, this study did not directly validate these explanatory mechanisms, and further exploration is needed in the future. For this subgroup, surgery may provide a more reliable local control advantage, and organ-preservation strategies should be considered cautiously. In addition, owing to the complexity of radical surgery, the efficacy of surgical treatment for upper thoracic esophageal cancer remains controversial. In our subgroup analysis, patients with upper thoracic esophageal cancer showed numerically higher 3-year PFS and OS rates in the RT group than in the surgery group, with absolute differences of 21.4% and 12.2%, respectively. However, these differences did not reach statistical significance.Given that upper thoracic tumors may exhibit robust regression following NICT (17), RT-based organ preservation could be a potential treatment option for selected patients, particularly responders, but this hypothesis requires validation in prospective studies with larger sample sizes. In conclusion, precise post-NICT response assessment is crucial for guiding subsequent therapeutic decisions. Emerging modalities, such as minimal residual disease monitoring and PET/CT examination, hold promise for identifying optimal candidates for organ preservation (18, 19). However, given the exploratory nature and limited statistical power of the subgroup analysis in the present study, the observed trends should be interpreted with caution.

Regarding safety, definitive RT following NICT demonstrated a manageable toxic profile. Although the incidence of grade 3 or higher toxicities during RT reached 75%, these were predominantly hematological, specifically lymphopenia (58.3%) and myelosuppression (18.3%), which is consistent with the findings of Song et al. (6). However, it was not possible to clearly attribute these toxicities to RT, prior NICT, or concurrent chemo-immunotherapy. Furthermore, compared with surgery, RT was not associated with a significant increase in treatment-related mortality (6, 20). Kong et al. (21) retrospectively analyzed the adverse reactions of RT after NICT in patients with ESCC and found that the incidence of grade 5 adverse events was as high as 14.2%, including esophageal fistula in 10.7% (6/56) and radiation pneumonitis in 3.6% (2/56). Although only one treatment-related death (1.7%) due to esophageal fistula was observed in our cohort, it is still necessary to carefully assess the toxicity of non-surgical treatment after NICT and be alert to the radiation damage that may be enhanced by immunotherapy (22).

In our study, locoregional recurrence exceeded 50% in both groups, although with divergent differences. In the surgery group, regional lymph node recurrence predominated (50.0%), a failure pattern likely attributable to residual micrometastases situated beyond the boundaries of standard lymphadenectomy (23, 24). Conversely, the RT group was characterized primarily by local esophageal recurrence (37.0%), consistent with failure patterns reported in the literature (21). Stratified analysis revealed similar trends among non-responders, which may be due to the fact that non-responders accounted for the highest proportion of patients with recurrence. However, an inverse trend emerged among the responders: the rate of locoregional recurrence was markedly lower in the RT group than in the surgery group (22.2% vs. 64.3%), whereas distant metastasis was the dominant failure mode in the RT group. Beyond the sample size limitations, we postulated that biological mechanisms underpin this observation. The status of near-cCR or cPR reflects the intrinsic tumor sensitivity to NICT. Furthermore, RT can induce immunogenic cell death (ICD) (25), which may synergize with memory T-cells generated by prior immunotherapy to elicit an adaptive immune response, thereby enhancing local control (26). While the utility of adjuvant therapy remains debated (27, 28), maintenance immunotherapy may be critical for this subgroup of responders. Extending the duration of therapeutic exposure may effectively suppress micrometastases, potentially reducing the distant failure rates. In conclusion, recurrence patterns vary significantly across different neoadjuvant modalities (24, 29). Given the complex underlying biology, future research should elucidate the specific mechanisms of tumor microenvironment remodeling and immune escape driving these recurrences.

This study has several limitations: 1) as a single-center retrospective study, residual confounding cannot be fully excluded despite the PSM analysis. Treatment allocation after NICT was not randomized and may have been influenced by tumor location, resectability, patient fitness, comorbidities, and patient preference, thereby introducing selection bias; 2) the absence of standardized guidelines for adjuvant or consolidation therapies following surgery or definitive RT may have contributed to heterogeneity in survival outcomes; 3) longer follow-up is needed to assess long-term survival and late-onset radiation toxicities; 4) the relatively small number of patients in the definitive RT group may have limited statistical power, particularly in subgroup analysis; 5) CT-based near-cCR may not fully correspond to pathological complete response or microscopic residual disease. Future multicenter, prospective randomized controlled trials with larger sample sizes are needed to validate these results.

## 5 Conclusions

Radical surgery remains the standard treatment for LA-ESCC after NICT. Definitive RT may be an organ-preserving option in selected patients, especially those with upper esophageal cancer or a favorable response to NICT.

## Data Availability

The data analyzed in this study is subject to the following licenses/restrictions: The datasets used and/or analysed during the current study are available from the corresponding author on reasonable request. Requests to access these datasets should be directed to Zhiguo Zhou, 47700723@hebmu.edu.cn.
