# Supplementary material for: Definitive radiotherapy versus surgery after neoadjuvant immunochemotherapy for locally advanced esophageal squamous cell carcinoma: a propensity score-matched study of efficacy and safety
Source: Front Oncol. 2026 Oct 12;16:1885302. doi: 10.3389/fonc.2026.1885302 (PMC13696280; doi:10.3389/fonc.2026.1885302)
Supplement: Supplementary file 1 [file Table1.docx]

**Table S1：Standardized mean difference data for each group before and after matching**

| **Variable** | **SMD before PSM** | **SMD after PSM** |
| --- | --- | --- |
| Propensity score distance | 0.53324090 | 0.01097011 |
| Age | 0.23218061 | 0.03363364 |
| Tumor location | 0.05695246 | 0.01072802 |
| Tumor length | 0.05593413 | 0.01653638 |
| cT stage | 0.10816338 | 0.02051700 |
| cN stage | 0.12341808 | 0.02277358 |
| Response to NCT | 0.46286794 | 0.02256624 |

SMD: Standardized mean differences

**Table S2 Univariate and multivariate Cox regression analysis of factors influencing PFS in the in the propensity score-matched cohort.**

| Variable | Univariate analysis | | Multivariate analyze | |
| --- | --- | --- | --- | --- |
|  | HR (95%CI) | *P*-Value | HR (95%CI) | P-value |
| Age |  | 0.382 |  |  |
| ≤65 | 1.223(0.779-1.921) |  |  |  |
| ＞65 | Ref |  |  |  |
| Sex |  | 0.653 |  |  |
| Male | 1.123(0.679-1.857) |  |  |  |
| Female | Ref |  |  |  |
| BMI |  | 0.714 |  |  |
| ≤23.15 | 1.088(0.694-1.706) |  |  |  |
| ＞23.15 | Ref |  |  |  |
| Tumor location |  | 0.736 |  | 0.580 |
| Ut | 0.827(0.410-1.670) | 0.597 | 0.813(0.389-1.702) | 0.584 |
| Mt | 1.081(0.659-1.771) | 0.759 | 1.176(0.709-1.951) | 0.529 |
| Lt | Ref |  | Ref |  |
| Tumor length |  | 0.971 |  | 0.845 |
| ≤5cm | 0.992(0.632-1.556) |  | 0.954(0.597-1.526) |  |
| ＞5cm | Ref |  | Ref |  |
| cT stage |  | 0.851 |  | 0.746 |
| T2 | 0.791(0.164-3.808) | 0.770 | 2.114(0.151-29.621) | 0.578 |
| T3 | 0.990(0.242-4.048) | 0.989 | 2.246(0.276-18.275) | 0.449 |
| T4a | Ref |  | Ref |  |
| cN stage |  | 0.002 |  | 0.804 |
| N0 | 0.350(0.190-0.647) | 0.001 | 0.536(0.083-3.464) | 0.513 |
| N1 | 0.799(0.465-1.372) | 0.415 | 0.900(0.494-1.639) | 0.730 |
| N2-3 | Ref |  | Ref |  |
| cTNM stage |  | 0.002 |  | 0.786 |
| II | 0.405(0.141-1.166) | 0.094 | 0.528(0.461-0.042) | 0.528 |
| III | 0.952(0.343-2.648) | 0.926 | 0.605(0.130-2.812) | 0.522 |
| IVA | Ref |  | Ref |  |
| Cycles of NICT |  | 0.897 |  |  |
| 2 | 1.031(0.652-1.630) |  |  |  |
| 3 | Ref |  |  |  |
| Response to NICT |  | 0.002 |  | 0.004 |
| near-cCR | 0.211(0.076-0.584) | 0.003 | 0.249(0.089-0.701) | 0.008 |
| cPR | 0.552(0.332-0.918) | 0.022 | 0.531(0.311-0.907) | 0.020 |
| cSD | Ref |  | Ref |  |
| Treatment modality |  | 0.120 |  | 0.089 |
| Surgery | 0.694(0.438-1.100) |  | 0.660(0.409-1.065) |  |
| RT | Ref |  | Ref |  |

**Table S3 Univariate and multivariate Cox regression analysis of factors influencing OS in the in the propensity score-matched cohort.**

| Variable | Univariate analysis | | Multivariate analyze | |
| --- | --- | --- | --- | --- |
|  | HR (95%CI) | *P*-Value | HR (95%CI) | P-value |
| Age |  | 0.146 |  |  |
| ≤65 | 1.476(0.873-2.493) |  |  |  |
| ＞65 | Ref |  |  |  |
| Sex |  | 0.582 |  |  |
| Male | 1.177(0.659-2.103) |  |  |  |
| Female | Ref |  |  |  |
| BMI |  | 0.738 |  |  |
| ≤23.15 | 1.094(0.647-1.849) |  |  |  |
| ＞23.15 | Ref |  |  |  |
| Tumor location |  | 0.814 |  | 0.475 |
| Ut | 1.008(0.453-2.245) | 0.984 | 0.872(0.375-2.028) | 0.751 |
| Mt | 1.190(0.661-2.144) | 0.562 | 1.352(0.738-2.479) | 0.329 |
| Lt | Ref |  | Ref |  |
| Tumor length |  | 0.690 |  | 0.661 |
| ≤5cm | 0.899(0.531-1.520) |  | 0.883(0.507-1.539) |  |
| ＞5cm | Ref |  | Ref |  |
| cT stage |  | 0.878 |  | 0.728 |
| T2 | 0.671(0.135-3.326) | 0.625 | 2.991(0.192-46.706) | 0.435 |
| T3 | 0.700(0.170-2.885) | 0.622 | 2.253(0.258-19.645) | 0.462 |
| T4a | Ref |  | Ref |  |
| cN stage |  | 0.012 |  | 0.653 |
| N0 | 0.347(0.173-0.695) | 0.003 | 0.559(0.078-3.669) | 0.562 |
| N1 | 0.627(0.336-1.167) | 0.141 | 0.724(0.359-1.463) | 0.368 |
| N2-3 | Ref |  | Ref |  |
| cTNM stage |  | 0.033 |  | 0.605 |
| II | 0.336(0.114-0.988) | 0.048 | 0.357(0.028-4.528) | 0.426 |
| III | 0.646(0.229-1.824) | 0.409 | 0.448(0.091-2.205) | 0.323 |
| IVA | Ref |  | Ref |  |
| Cycles of NICT |  | 0.653 |  |  |
| 2 | 0.886(0.521-1.505) |  |  |  |
| 3 | Ref |  |  |  |
| Response to NICT |  | 0.006 |  | 0.010 |
| near-cCR | 0.149(0.036-0.619) | 0.009 | 0.174(0.041-0.734) | 0.017 |
| cPR | 0.521(0.283-0.957) | 0.036 | 0.498(0.260-0.956) | 0.036 |
| cSD | Ref |  | Ref |  |
| Treatment modality |  | 0.031 |  | 0.019 |
| Surgery | 0.557(0.328-0.947) |  | 0.514(0.295-0.898) |  |
| RT | Ref |  | Ref |  |
